# Phenotypic diversity identified by cardiac magnetic resonance in a large hypertrophic cardiomyopathy family with a single MYH7 mutation

**DOI:** 10.1038/s41598-018-19372-4

**Published:** 2018-01-17

**Authors:** Jie Wang, Ke Wan, Jiayu Sun, Weihao Li, Hong Liu, Yuchi Han, Yucheng Chen

**Affiliations:** 1 Department of Cardiology, West China Hospital, Sichuan University, Chengdu, Sichuan 610041 China; 2 Department of Radiology, West China Hospital, Sichuan University, Chengdu, 610041 Sichuan Province P. R. China; 3 Department of Medicine (Cardiovascular Division), University of Pennsylvania, Philadelphia, Pennsylvania USA

## Abstract

Limited data is available on phenotypic variations with the same genotype in hypertrophic cardiomyopathy (HCM). The present study aims to explore the relationship between genotype and phenotype characterized by cardiovascular magnetic resonance (CMR) in a large Chinese family. A proband diagnosed with HCM from a multigenerational family underwent next-generation sequencing based on a custom sureSelect panel, including 117 candidate pathogenic genes associated with cardiomyopathies. All genetic results were confirmed by the Sanger sequencing method. All confirmed mutation carriers underwent CMR exam and myocardial tissue characterization using T1 mapping and late gadolinium enhancement (LGE) on a 3T scanner (Siemens Trio, Gemany). After clinical and genetic screening of 36 (including the proband) members of a large Chinese family, nineteen family members are determined to carry the single p.T1377M (c.4130C>T) mutation in the MYH7 gene. Of these 19 mutation carriers, eight are diagnosed with HCM, one was considered as borderline affected and ten are not clinically or phenotypically affected. Different HCM phenotypes are present in the nine affected individuals in this family. In addition, we have found different tissue characteristics assessed by T1 mapping and LGE in these individuals. We describe a family that demonstrates the diverse HCM phenotypes associated with a single MYH7 mutation.

## Introduction

Hypertrophic cardiomyopathy (HCM) is an autosomal dominant genetic disease where nearly 60% of the disease is caused by mutations of myocardial sarcomere proteins. Of those, myosin heavy chain beta isoform (MYH7) mutations account for 35~50%^1^, especially the mutations located in the head and neck domains of the MYH7 gene^2^. More than 200 mutations were described in MHY7^3^ and different mutations could lead to other cardiomyopathies, including restrictive cardiomyopathy (RCM), dilated cardiomyopathy (DCM), and left ventricular non-compaction (LVNC)^3^. In addition, MYH7 with different point mutations could lead to different clinical manifestations and disease progression. For instance, HCM patients with Arg453Cys mutation in the MYH7 gene might have a higher incidence of end stage heart failure and premature death^4^. Similarly, Gly716Arg, Arg719Trp, Arg719Gln, Arg723Gly, or Gly741Arg mutations have malignant clinical manifestations^5–8^. On the contrary, patients with MYH7 point mutation of Val606Met have a good prognosis^9^. Due to the autosomal dominant inheritance pattern of HCM, familial forms which result from a single genetic mutation with a Mendelian inheritance pattern are increasingly recognized^10^. There are a few case reports of families with single MYH7 mutations that have diverse clinical manifestations and disease progression. For instance, Arg787His^11^ or Gly425Arg^12^ mutation in MYH7 gene showed significant differences in clinical manifestations.

Recently, targeted gene panels based on next-generation sequencing (NGS) method has been validated in multiple studies^13,14^ and they are expected to provide increased depth of coverage with higher sensitivity and specificity by using a limited number of genes to detect genetic changes in patients with cardiomyopathies^15,16^.

Cardiovascular magnetic resonance (CMR) is considered the reference noninvasive standard for the evaluation of left ventricular (LV) volumes, ejection fraction (EF), mass, and tissue characterization using T1 mapping and late gadolinium enhancement (LGE). CMR has been increasingly utilized as the standard imaging modality for HCM patients. However, scarce data exists on the genotype–phenotype association in patients with HCM as assessed by CMR. In this report, we describe a large HCM family with a single MYH7 mutation and diverse HCM phenotypes as characterized by CMR.

## Methods

### Clinical evaluation

We conducted clinical evaluations which consisted of family history, previous medical history, physical examination, 12-lead electrocardiography (ECG), and echocardiography. Contrast enhanced chest computed tomography (CT) and digital subtraction angiography (DSA) were performed when necessary for clinical diagnosis. Diagnosis of HCM was made according to the AHA/ACC guideline^17^, and all methods were performed in accordance with the approved guidelines. This study was approved by the ethics committee of West China Hospital, and written informed consents were obtained from all participants.

## Genotyping

The screening genetic panel included 117 candidate genes reported to be causative of cardiomyopathy, according to Online Mendelian Inheritance in Man (http://omim.org) and PubMed literature review. The gene list is shown in Supplemental Table S1. After obtaining informed consent, genomic DNA was extracted and captured with TruSeq DNA sample preparation kit and sequenced on a Hiseq platform. Genetic results were confirmed by the Sanger sequencing method subsequently. Pathogenicity determination of gene mutation was performed following guideline recommendations^18^.

### Image acquisition and analyses

We obtained echocardiographic parameters according to the American College of Cardiology/American Heart Association guidelines^19^, including measurements of cardiac chamber dimensions, ventricular wall thickness, and measurements of LVEF.

ECG gated CMR imaging was performed on a 3.0-T Magnetom scanner (Trio Tim; Siemens Healthineers, Erlangen, Germany) with a with a 32-channel dedicated cardiac phase-array receiver coil. SSFP cine images of the LV from the base to apex in consecutive short-axis views and long-axis views (two-, three-, and four-chamber) were acquired during breath-holds with the following parameters: temporal resolution: 42 ms; repetition time (TR), 3.4ms; echo time (TE), 1.3ms; flip angle (FA), 50 degrees; field of view, 320–340 mm; matrix size, 256 × 144; Reconstructed in plane spatial resolution was 1.4 mm*1.3 mm and slice thickness of 8 mm with no gap. Native T1 measurements were acquired at the basal, mid, and apical short axis before injection of gadolinium using motion corrected modified Look-Locker inversion recovery sequence (MOLLI) (Siemens Healthcare works-in-progress 448; protocol: 5(3)3). Parameters for MOLLI was as follows: TR 740ms, TE 1.06ms, FA 35°, bandwidth 930 Hz/pixel, initial T1 100ms with 80ms increments, parallel imaging factor 2, matrix 192*144, in plane spatial resolution 2.4*1.8 mm, total acquisition time 11 heart beats. T1 measurements were repeated in the identical short-axis slices at about 15 minutes after administration of gadolinium by (MOLLI protocol: 4(1)3(1)2). Hematocrit was tested within 24 hours of CMR scanning for extracellular volume (ECV) calculation.

LGE images were acquired at 10–15 minutes after intravenous administration of 0.15 mmol/kg of gadopentetate dimeglumine (Magnevist, Bayer Schering Pharma, Berlin, Germany) using the inversion recovery method with phase-sensitive reconstruction (PSIR) on identical short and long axis views: TR 700 ms; TE 1.56 ms; FA 20°; matrix 256 × 144; inversion time (TI) was individually optimized to null normal myocardial signal using a TI scout sequence.

LV volumes, EF, and mass were analyzed using commercially available software (Qmass 7.6; Medis Medical Imaging Systems, Leiden, the Netherlands) by two experienced observers. LV endocardial and epicardial borders on cine images were manually drawn to define the myocardium, taking care to exclude papillary muscles and the trabeculations from the blood pool. Maximal LV wall thickness was defined as the greatest dimension anywhere within the LV myocardium. LGE is defined quantitatively by a myocardial signal intensity of 6 standard deviations from the normal myocardium and the amount of LGE is semi-automatically quantified using the QMASS 7.6 software. Total LGE was expressed as a proportion of LV myocardium (%LGE).

To quantify T1 values, the endocardial and epicardial contours were traced manually with care not to include trabeculations, blood pool or epicardial fat on the pre-contrast and post-contrast T1-mapping images at the mid LV slice, including regions that were positive for LGE^20,21^. After fitting the curve using the QMass7.6 software, mean myocardial T1 values was obtained. Blood T1 was obtained by locating a region of interest in the blood pool within the LV cavity in the corresponding pre-contrast and post-contrast T1-mapping images. The ECV was calculated as follow: ECV = (1−HCT) * ([1/T1myo post-1/T1myo pre]/[1/T1blood post-1/T1blood pre]).

## Results

### Clinical characteristics and phenotypes

**Figure 1:**
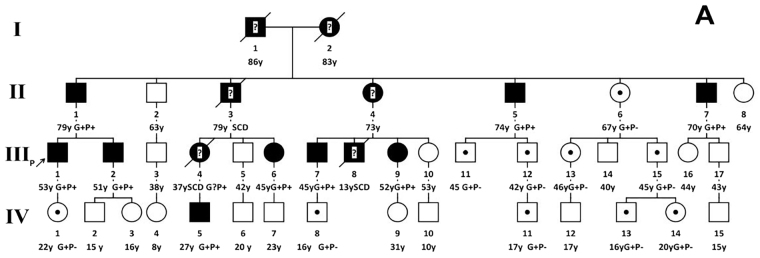
Pedigree of the family. Squares represent male relatives; circles represent female relatives; filled symbols indicate HCM patients; slants indicate dead members; arrow represent proband; symbol with dots represent mutation carrier with negative phenotype; ? represent that the subject died before our investigation so that we cannot get clinical data and confirm their phenotype; SCD: Sudden cardiac death: G+: positive genotype; P+: positive phenotype; P−: negative phenotype. IV5 was considered as borderline affected with a maximal LV thickness of 13.2 mm.

**Figure 2:**
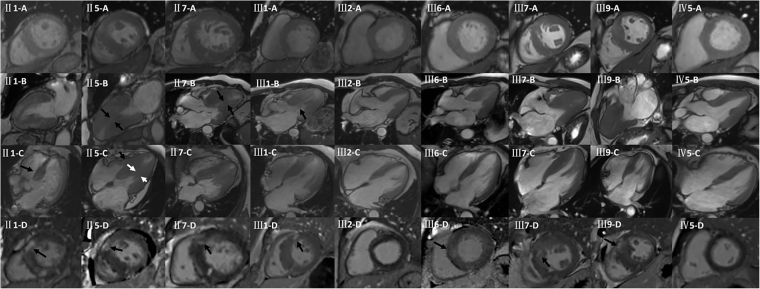
Cardiac morphologies evaluated by CMR; II1 had sigmoid septum (black arrow of II1-C points to basal septal hypertrophy) without LVOT obstruction; II5 had the neutral septum (II5-C: white arrow)with RV involvement (II5-C: black arrow); II7 had neutral septum(II7-B: black arrow) with no RV involvement; III1 had sigmoid septum with LVOT obstruction (III1**-**B:black arrow); III2 had sigmoid septum without LVOT obstruction; III6 had sigmoid septum without LVOT obstruction; III7 had reversed curvature of the septum; III9 had reversed curvature of the septum; IV5 had symmetric LV hypertrophy; Black arrows of II1-D, II5-D, II7-D, III1-D, III6-D, III7-D, and III9-D point to LGE in the myocardium.

The pedigree of the proband and 35 family members is shown in Fig. 1. The proband (III1) was a 51 years old man who experienced palpitation for 9 years, and had chest pain and mild dyspnea on exertion for two years before admission to our hospital. On admission, the patient presented with a slightly elevated cardiac troponin T level of 21.8 ng/L (normal range 0 to 14 ng/L) and a significantly elevated N-terminal pro-brain natriuretic peptide level of 537 pg./mL (normal range 0 to 125 pg/mL). ECG showed supraventricular tachycardia and non-specific ST-T changes. Coronary angiography showed no significant coronary artery disease. Echocardiography showed significant left atrial enlargement, preserved biventricular systolic function, and severe LV outflow tract obstruction (peak aortic outflow velocity = 4.8 m/s, peak gradient = 92 mmHg). The abnormal morphology and function were confirmed by CMR, which showed a sigmoid septum with LV outflow tract obstruction (Fig. 2III1-B,C) and LV mass index to body surface area (BSA) of 87.2 g/m^2^ (papillary muscles excluded). The maximal thickness was 23.7 mm on CMR. In addition, CMR also showed LGE in the interventricular septum (Fig. 2III1-D). The proband was successfully managed medically with ß-adrenergic receptor blocker and diuretics.

The family included a total of 42 members and five were deceased. One member was not able to be genotyped. Eighteen of the 35 family members (not including the proband) were found to carry the same MYH7 gene mutation and underwent CMR scan. Of those 18 family mutation carriers, 7 were diagnosed with HCM, 1 (IV5) was considered as borderline affected with a maximal LV thickness of 13.2 mm, and 10 were found to not be affected. Details of the demographic, clinical characteristics, and CMR parameters including tissue characteristics of the 8 (including the proband) HCM family members and 1 borderline affected member are shown in Table 1, whereas 10 phenotypically negative mutation carriers are shown in Table 2. In addition, three deceased members (II3, III4, III8) experienced cardiac sudden death by history. Subjects II3 and III8 were asymptomatic without medical history prior to their deaths. Subject II3 died suddenly at age 79 while doing morning exercise, whereas subject III8 died suddenly at age 13 while playing basketball. Subject III4 experienced severe dyspnea on exertion for 1 year and was treated with septal myomectomy at an outside hospital. She succumbed to sudden death 18 months after surgery at age 37. Other family members’ clinical examination, ECG, echocardiography and genetic analyses were normal and were considered unaffected.

**Table 1:** Demographic, clinical and CMR characteristics in 8 HCM family members and one borderline affected subject (IV5) with the single MYH7 mutation.

Subject	Age	Sex	Symptoms	Syncope	Cardiac phenotype	ECG	Max LVT (mm)	LV EF (%)	%LGE	Native T1(ms)	ECV (%)	Clinical course
II1	79	M	Chest pain	Yes	Sigmoid septum, no-obstruction	NSR, PVC detected by 24 h Holter	17.1	42.5%	38.5	1287.0	30.8	Heart failure managed with BB
II5	74	M	Palpitation	Yes	Neutral septum, RV involvement	NSR, NSST-T	30.7	55.4%	21.6	1233.0	29.0	ICD implantation
II7	70	M	None	No	Neutral septum, no RV involvement	NSR, NSST-T	28.0	54.6%	16.3	1167.0	27.6	Medically managed with BB
III1	53	M	Palpitation, chest pain, DOE	No	Sigmoid septum with obstruction	NSR, NSST-T, PSVT detected by 24 h Holter	23.7	63.7%	6.0	1278.6	27.0	Medically managed with BB
III2	51	M	None	No	Sigmoid septum, no-obstruction	NSR	16.4	57.5%	0	1183.9	24.7	Medically managed with BB
III6	46	F	None	No	Sigmoid septum, no-obstruction	NSR	16.8	72%	5.7	1271.0	28.1	Medically managed with BB
III7	45	M	Chest pain, DOE	No	Reversed curvature	NSR,NSST-T	17.2	59.9%	11.0	1170.3	27.4	Medically managed with BB
III9	52	F	Chest discomfort, NYHA II	No	Reversed curvature	NSR	24.0	68%	8.0	1235.1	28.3	Heart failure managed with BB and diuretics
IV5	27	M	None	No	Symmetric hypertrophy	NSR	13.2	54.2%	0	1216.1	26.8	Medically managed with BB.

M = male, F = female; EF; Ejection fraction; LVT: left ventricular thickness; LGE: Late gadolinium enhancement; ECG: Electrocardiogram; PVC: Premature ventricular contractions; PSVT: Paroxysmal supraventricular tachycardia: NSST-T: Nonspecific ST-T changes; NSR: Normal sinus rhythm, DOE: Dyspnea on exertion; LV = Left ventricle; RV = Right ventricle; ECV = Extracellular volume; BB = β-adrenergic receptor blocker.

**Table 2:** Demographic, clinical and CMR- characteristics in 10 phenotypically negative mutation carrier family members.

Subjects	Age	Sex	Symptoms	ECG	LVT max (mm)	LV EF (%)	RV EF (%)	%LGE	Native T1(ms)	ECV (%)
II6	67	F	None	NSR	12.0	64.5	63.2	0	1200.5	27.7
III11	45	M	None	NSR	11.0	64.0	65.0	0	1152.5	27.4
III12	42	M	None	NSR	8.0	60.0	59.4	0	1174.3	26.6
III13	46	F	None	NSR	9.0	66.3	66.7	0	1136.9	25.3
III15	45	M	None	NSR	8.0	68.5	64.5	0	1116.0	25.6
IV1	22	F	None	NSR	8.2	60.2	58.6	0	1249.1	25.4
IV8	16	M	None	NSR	9.0	70.2	68.5	0	1124.5	27.0
IV11	17	M	None	NSR	8.4	65.2	63.2	0	1199.9	27.0
IV13	16	M	None	NSR	9.5	69.2	68.5	0	1177.3	28.0
IV14	20	F	None	NSR	8.2	60.2	58.5	0	1262.1	28.6

M = male, F = female; EF; Ejection fraction; LVT: left ventricular thickness; LGE: Late gadolinium enhancement; ECG: Electrocardiogram.

NSR:Normal sinus rhythm; LV = Left ventricle; RV = Right ventricle; ECV = Extracellular volume.

### Genotype-phenotype investigations

**Figure 3:**
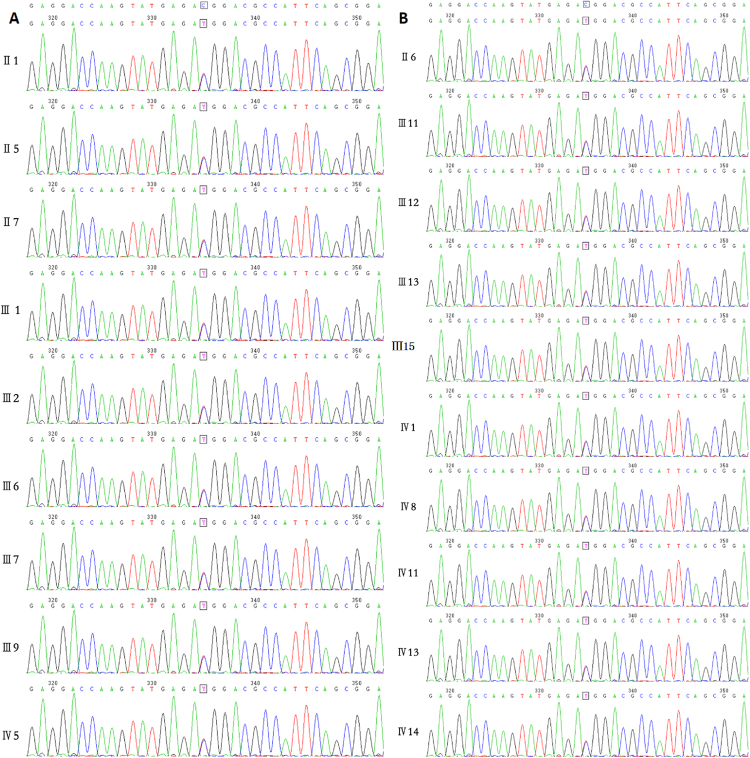
(**A**) Mutation in MYH7 gene for the proband III1, his other 7 family members with positive phenotype and one borderline affected subject (p.T1377M, c.4130C > T). (**B**) Mutation in MYH7 gene for other 10 family members of the proband with negative phenotype (p.T1377M, c.4130C > 44T).

Genotyping confirmed that the family had a mutation in the MYH7 gene. The proband (III1, Fig. 3) and the other 18 family members (Fig. 3) was identified to carry the p.Thr1377Met (c.4130C > T) mutation in MYH7 gene confirmed by the Sanger method. Of the 18 mutation carrier family members, ten proved to be clinically not affected because they experienced no symptoms, and had normal echocardiograms and normal CMR findings (Supplemental Figs 1 and 2). Eight individuals (including the proband) diagnosed with HCM and one borderline affected subject had different HCM phenotypes, including the sigmoid septum with and without LVOT obstruction, neutral septum with right ventricular (RV) involvement, neutral septum without RV involvement, reversed curvature, and symmetrical hypertrophy phenotypes (Fig. 2).

### T1 mapping and LGE assessment by CMR

Native T1 ranged from 1167 ms to 1287.0 ms and ECV ranged from 24.7% to 30.8% in the phenotypically affected individuals (Table 1). Different degrees of fibrosis in different location of the myocardium were identified by LGE in the phenotypically positive subjects. Native T1 (ranging from 1116.0 to 1262.1 ms) and ECV (ranging from 25.3% to 28.6%) were also found in the 10 phenotypically negative mutation carriers (Table 2).

## Discussion

In this study, we report the diverse phenotypes with a single point mutation in MYH7 in a large HCM family. More than half of the mutation carriers were phenotypically negative for HCM. The phenotypically positive HCM subjects exhibited a variety of HCM phenotypes.

The clinical features of the family is interesting for several reasons. First, a single p.Thr1377Met mutation in the MYH7 gene is shown to induce different phenotypes of HCM or not to induce any abnormal phenotype at all. Although the single point mutation had been previously reported in HCM^2^, we are the first to report the variable expression in a large family. Second, HCM patients carrying the same point mutation may have different clinical manifestations. The subject II3 had a near normal life span before sudden death but III8 experienced sudden death at a very young age. III4 experienced sudden death18 months after surgical septal myectomy and II5 was recommended to have an ICD implantation. Third, previous works showed that areas of focal fibrosis identified by LGE was associated with prognosis in HCM^22^, diffuse fibrosis detected by ECV had significant association to fibrosis measured histologically^23–25^, and different morphological subtype of hypertrophic cardiomyopathy could have diverse prognosis^26–28^. In the present study, the 8 HCM phenotypically positive and 1 borderline affected individuals had variable morphological subtypes, focal fibrosis by LGE, and diffuse fibrosis by ECV, which provided strong evidence for variable phenotypes associated with the same genotype. Fourth, the mutation carriers also had a range of native T1 and ECV values in the absence of LGE, which indicated phenotypically negative mutation carriers may have abnormal tissue characteristics as compared to healthy controls^29^. However, the implication of this for the genotype positive phenotype negative carriers is unknown and future research is needed.

Little data exists describing the genotype and CMR-phenotype relationship in HCM patients. Most of the research focused on the comparison of different phenotypes and prognosis caused by different genetic mutations in HCM^3,30,31^, or the difference between the HCM patients and genetic carriers with negative phenotype^32–34^. There were reports that described a single site mutation in the MYH7 gene producing not only a classic hypertrophic pattern but also a RCM phenotype^35–38^. Despite early hopes that genotyping may predict cardiac morphology and long-term outcomes in HCM, dedicated studies have failed to support this hypothesis^39^. In addition, there were large families with the same point mutation, which was reported to have 60% penetrance with different degree of LV hypertrophy and clinical presentation from asymptomatic to sudden death^40^. Genotype may not be an effective way to prognosticate or determine the degree of severity of disease expression of certain genes. Our findings provide imaging evidence that some genotypes may not be useful for the prediction of cardiac phenotypes in HCM.

It is unclear why the same mutation could produce diverse phenotypes. As for the single MYH7 mutation induced phenotypic diversity in different HCM individuals^11^, additional genetic modifiers could provide an explanation for different phenotypes. It suggested that there might be other factors regulating the expression of pathogenic mutations in the course of disease^41^. Of HCM modifying genes, such as angiotensin-converting enzyme (ACE)-1, endothelin-1, and tumor necrosis factor-α, their function had not been systematically studied. However, single nucleotide polymorphism (SNPs) and the relationship between the disease progression of HCM and the severity of phenotype has been studied. For example, ACE-1 had at least 13 SNPs. Relative studies had confirmed that the ACE-1 or ACE-2 mutation was associated with the severity of LV hypertrophy in HCM^42,43^. In addition, other genes such as myosin binding protein H have been investigated as modifiers of the hypertrophic variability in HCM^44^. Furthermore, complex genetic or a complex hierarchy of genetic, epigenetic, and environmental factors (such as demographic factors including age, sex, and body size, hypertension or obesity) might be involved in determining the phenotype^40^. In addition to pathogenic genes, background genes and methylation modification might play a very important role on HCM phenotype^45–47^.

Genetic testing in patients with HCM has been used to exclude differential diagnoses including Fabry disease and screen family to identify high risk members. Genetic testing has not been used in the treatment of HCM. Our study adds further evidence that our current understanding of genotype may be insufficient to predict phenotype or outcomes. We need to further explore the mechanisms leading to the diverse phenotypes and identifying high-risk markers for the development of hypertrophy. Because the occurrence of HCM may involve multiple independent signaling pathways, it is important to clarify the specific signaling pathways to effectively treat each subtype of HCM.

The relationship between the genotype, phenotype, and prognosis need further follow-up studies. In addition, the mechanism involved in the different phenotypes still need be explored.

In summary, we report a large Chinese families carrying a single MYH7 (p.Thr1377Met, c.4130C > T) genetic mutation which is associated with both the absence of HCM phenotype and diverse phenotypes of HCM. Additional studies are needed to clarify the mechanisms.

## Electronic supplementary material

Supplementary Information
