# Supplementary material for: Phenotypic diversity identified by cardiac magnetic resonance in a large hypertrophic cardiomyopathy family with a single MYH7 mutation
Source: Sci Rep. 2018 Jan 17;8:973. doi: 10.1038/s41598-018-19372-4 (PMC5772531; doi:10.1038/s41598-018-19372-4)

Phenotypic diversity identified by cardiac magnetic resonance in a large hypertrophic cardiomyopathy family with a single MYH7 mutation

Jie Wang, BMSci[a†], Ke Wan, BMSci [a†] ,Jiayu Sun, MD[b] , Weihao Li, BMSci[a] ,Hong Liu, BMSci[a], Yuchi Han, MD, MMSc, FACC[c], Yucheng Chen, MD, FACC[*a]

†Both authors contributed equally to this work

[a] Department of Cardiology, West China Hospital, Sichuan University, Chengdu, Sichuan, 610041, China

[b] Department of Radiology, West China Hospital, Sichuan University, Chengdu 610041, Sichuan Province, P. R. China

[c] Department of Medicine (Cardiovascular Division), University of Pennsylvania, Philadelphia, Pennsylvania, USA

Corresponding author:

Yucheng Chen, MD, FACC

Professor, Department of Cardiology,

West China Hospital, Sichuan University, Guoxue Xiang No. 37, Chengdu, Sichuan, 610041, China

Tel: 86-18980602149

Fax: 86-028-85422836

E-mail: chenyucheng2003@126.com

Supplemental Table S1. List of the genes selected to perform the custom array.

Gene	mRNA	Chrs*	Segs(n)#	Seg（bp）&	Trans（n）¥
RRAGC	NM_001271851,NM_022157	chr1	8	3583	2
TMPO	NM_001032283,NM_001032284,NM_003276,NM_001307975	chr12	11	7993	4
RBM20	NM_001134363	chr10	15	8435	1
IDS	NM_001166550,NR_104128,NM_006123,NM_000202	chrx	11	7197	4
TGFB3	NM_001329938,NM_001329939,NM_003239	chr14	8	4308	3
IDUA	NR_110313,NM_000203	chr4	15	3418	2
KRAS	NM_004985,NM_033360	chr12	7	6689	2
HGD	NM_000187	chr3	15	3210	1
DOLK	NM_014908	chr9	2	2754	1
SYNE1	NM_182961,NM_001347702,NM_001347701,NM_033071	chr6	150	36288	4
CTNNA3	NM_013266,NM_001291133,NM_001127384	chr10	23	14469	3
SGSH	NM_000199	chr17	9	3668	1
EMD	NM_000117	chrx	7	2139	1
VCL	NM_003373,NM_014000	chr10	23	7086	2
MUT	NM_000255	chr6	14	5036	1
ANKRD1	NM_014391	chr10	10	2924	1
ALMS1	NM_015120	chr2	24	14572	1
LAMA4	NM_001105206,NM_001105208,NM_001105207,NM_001105209,NM_002290	chr6	40	10017	5
GUSB	NM_000181,NM_001293104,NM_001293105,NR_120531,NM_001284290	chr7	13	3399	5
NDUFS2	NM_004550,NM_001166159	chr1	16	3975	2
DTNA	NM_001198940,NM_001198941,NM_001198942,NM_001392,NM_001390,NM_001198939,NM_001391,NM_001198943,NM_001198944,NM_001198945,NM_032978,NM_032980,NM_001128175,NM_032975,NM_032979,NM_032981,NM_001198938	chr18	34	15055	17
DSG2	NM_001943	chr18	16	6903	1
CRYAB	NM_001330379,NM_001289808,NM_001289807,NM_001885	chr11	6	2283	4
FAH	NM_000137	chr15	15	2997	1
COX10	NM_001303	chr17	8	3852	1
DMD	NM_004010,NM_004006,NM_004014,NM_004016,NM_004013,NM_004021,N	chrx	92	23462	17

	M_000109,NM_004017,NM_004018,NM_004020,NM_004019,NM_004015,NM_004011,NM_004022,NM_004012,NM_004023,NM_004009				
MYH6	NM_002471	chr14	40	8391	1
MYH7	NM_000257	chr14	41	8555	1
ILK	NM_001014794,NM_001014795,NM_001278442,NM_004517,NM_001278441	chr11	13	3388	5
DMPK	NM_001081560,NM_001288764,NM_001288765,NM_001288766,NM_001081563,NM_001081562,NM_004409	chr19	18	5955	7
NDUFV2	NM_021074	chr18	9	1830	1
AGL	NM_000644,NM_000646,NM_000642,NM_000028,NM_000643	chr1	38	11908	5
FUCA1	NM_000147	chr1	9	3017	1
PDLIM3	NM_001114107,NM_001257963,NR_047562,NM_001257962,NM_014476	chr4	10	3990	5
LMNA	NM_170708,NM_001282626,NM_001282624,NM_005572,NM_001257374,NM_170707,NM_001282625	chr1	20	6454	7
GLA	NM_000169	chrx	8	2268	1
ARSB	NM_198709,NM_000046	chr5	11	8125	2
TNNT2	NM_001001430,NM_001001431,NM_001001432,NM_001276347,NM_001276346,NM_000364,NM_001276345	chr1	20	2797	7
NEBL	NM_213569,NM_001173484,NM_006393	chr10	34	12526	3
RYR2	NM_001035	chr1	106	22115	1
PKP2	NM_001005242,NM_004572	chr12	15	5639	2
MYBPC3	NM_000256	chr11	36	6474	1
AGA	NM_001171988,NM_000027,NR_033655	chr4	10	3052	3
ACTN2	NM_001278344,NM_001103,NM_001278343	chr1	24	6760	3
MIB1	NM_020774	chr18	22	11117	1
TMEM43	NM_024334	chr3	13	4442	1
LDB3	NM_001080114,NM_001171611,NM_001080116,NM_001171610,NM_001080115,NM_007078	chr10	17	8041	6
MYPN	NR_045663,NM_001256268,NM_001256267,NM_032578,NR_045662	chr10	28	8749	5
CASQ2	NM_001232	chr1	12	3750	1
TMEM70	NR_033334,NM_017866,NM_001040613	chr8	5	2892	3

LAMA2	NM_001079823,NM_000426	chr6	67	13498	2
DSC2	NM_004949,NM_024422	chr18	18	6604	2
SCO2	NM_005138,NM_001169109,NM_001169110,NM_001169111	chr22	6	2855	4
PYGM	NM_001164716,NM_005609	chr11	21	5116	2
PLN	NM_002667	chr6	3	2314	1
TNNC1	NM_003280	chr3	7	1496	1
GNPTAB	NM_024312	chr12	22	7180	1
ACAT1	NM_000019	chr11	13	3234	1
GNS	NM_002076	chr12	15	6328	1
AGK	NM_018238	chr7	17	4225	1
PTPN11	NM_001330437,NM_002834,NM_080601	chr12	17	7877	3
NKX2-5	NM_001166176,NM_004387,NM_001166175	chr5	3	2561	3
NAGA	NM_000262	chr22	10	4676	1
MYOZ1	NM_021245	chr10	7	2383	1
PSEN1	NM_007318,NM_000021	chr14	13	7190	2
JUP	NM_002230,NM_021991	chr17	15	4708	2
NAGLU	NM_000263	chr17	7	3582	1
EYA4	NM_004100,NM_172105,NM_172103,NM_001301012,NM_001301013	chr6	21	7492	5
GYS1	NM_001161587,NM_002103,NR_027763	chr19	17	4918	3
SCN5A	NM_001099405,NM_001160160,NM_001160161,NM_198056,NM_001099404,NM_000335	chr3	30	10548	6
TTN	NM_001256850,NM_133379,NM_133437,NM_003319,NM_001267550,NM_133432,NM_133378	chr2	365	135554	7
FXN	NM_001161706,NM_181425,NM_000144	chr9	7	8254	3
RAF1	NM_002880	chr3	18	4625	1
NRAS	NM_002524	chr1	8	5304	1
ANO5	NM_213599,NM_001142649	chr11	23	8261	2
GALNS	NM_001323544,NM_001323543,NM_000512	chr16	15	3563	3
CALR3	NM_145046	chr19	10	2242	1
TCF21	NM_198392,NM_003206	chr6	4	4304	2
ABLIM1	NM_001003407,NM_001003408,NM_001322894,NM_001322882,NM_001322896,NM_001322897,NM_001322898,NM_001322899,NM_001322892,NM_001322883,NM_002313,NM_001322885,NM_	chr10	29	11111	23

	001322893,NM_001322895,NM_006720,NM_001322886,NM_001322889,NM_001322884,NM_001322887,NM_001322891,NM_001322888,NM_001322890,NM_001322900				
HGSNAT	NM_152419	chr8	19	6614	1
SLC25A4	NM_001151	chr4	5	5120	1
POLG	NM_002693,NM_001126131	chr15	24	6098	2
RIT1	NM_001256821,NM_006912,NM_001256820	chr1	8	4818	3
COX15	NM_004376,NM_001320974,NM_078470,NM_001320976,NM_001320975	chr10	10	8639	5
MYL3	NM_000258	chr3	8	1779	1
GLB1	NM_001079811,NM_001135602,NM_001317040,NM_000404	chr3	18	4440	4
PSEN2	NM_012486,NM_000447	chr1	14	3448	2
DSP	NM_001008844,NM_004415,NM_001319034	chr6	25	11430	3
GBE1	NM_000158	chr3	17	4401	1
FHL1	NM_001330659,NM_001159700,NM_001159703,NM_001159704,NM_001167819,NM_001159699,NR_027621,NM_001449,NM_001159701,NM_001159702	chrx	16	7702	10
CAV3	NM_001234,NM_033337	chr3	3	2031	2
PRKAG2	NM_001040633,NM_001304527,NM_024429,NM_001304531,NM_016203	chr7	23	7756	5
CSRP3	NM_003476	chr11	8	2314	1
TPM1	NM_001018004,NM_001018006,NM_001018008,NM_001018020,NM_001330344,NM_001330346,NM_001330351,NM_000366,NM_001301289,NM_001301244,NM_001018007,NM_001018005	chr15	17	5099	12
HEXB	NM_000521,NM_001292004	chr5	17	4187	2
CTSA	NM_001127695,NM_001167594,NM_000308,NR_133656	chr20	16	3536	4
TNNI3	NM_000363	chr19	9	1754	1
ARSA	NM_001085425,NM_001085426,NM_001085428,NM_001085427,NM_000487	chr22	9	5215	5
MYL2	NM_000432	chr12	8	1679	1
BAG3	NM_004281	chr10	5	3269	1
LAMP2	NM_001122606,NM_002294,NM_013995	chrx	11	10388	3
TCAP	NM_003673	chr17	3	1563	1
GATAD1	NM_021167,NR_052016	chr7	6	5347	2

AGXT	NM_000030	chr2	12	2648	1
CTF1	NM_001330,NM_001142544	chr16	4	2314	2
GRHPR	NM_012203	chr9	10	2185	1
DNAJC19	NM_001190233,NR_033721,NM_145261,NR_033723,NR_033722,NR_046073	chr3	8	2746	6
ACTC1	NM_005159	chr15	8	4543	1
MYOZ2	NM_016599	chr4	7	3397	1
MYH7B	NM_020884	chr20	46	9334	1
DES	NM_001927	chr2	10	3198	1
NONO	NM_001145409,NM_001145408,NM_007363,NM_001145410	chrx	14	4368	4
TAZ	NM_181313,NM_000116,NM_181312,NR_024048,NM_181311,NM_001303465	chrx	12	3136	6
GAA	NM_000152,NR_134848,NM_001079803,NM_001079804	chr17	21	5275	4
SGCD	NM_000337,NM_001128209,NM_172244	chr5	10	11094	3
NEXN	NM_144573,NM_001172309	chr1	14	4539	2
ABCC9	NM_005691,NM_020297	chr12	40	10912	2

*chr: chromosome

#Segs (n): number of segments

&Segs (bp): length (bp) of the screened gene regions

¥Transc (n): Number of transcripts captured

Supplemental Figure 1. Cardiac morphologies were evaluated by CMR; Ⅱ6, Ⅲ11 , Ⅲ12, Ⅲ13, and Ⅲ15 showed normal CMR findings.

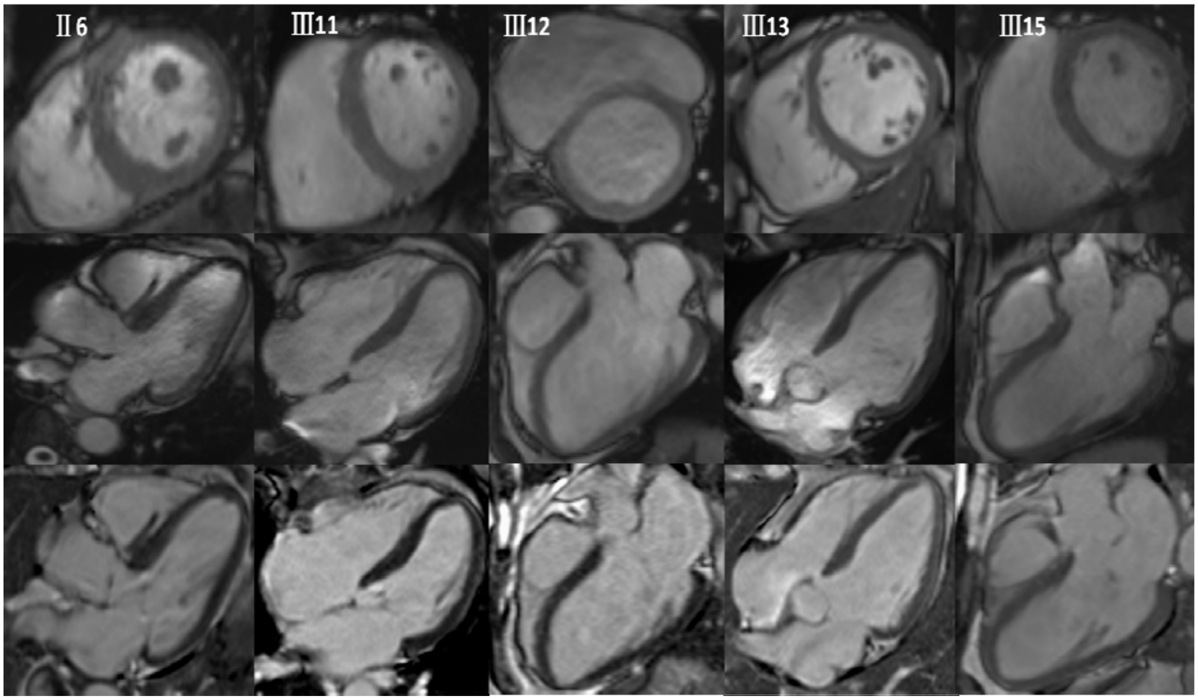

Supplemental Figure 2. Cardiac morphologies were evaluated by CMR; Ⅳ1, Ⅳ8, Ⅳ11, Ⅳ13, and Ⅳ14 showed normal CMR findings.

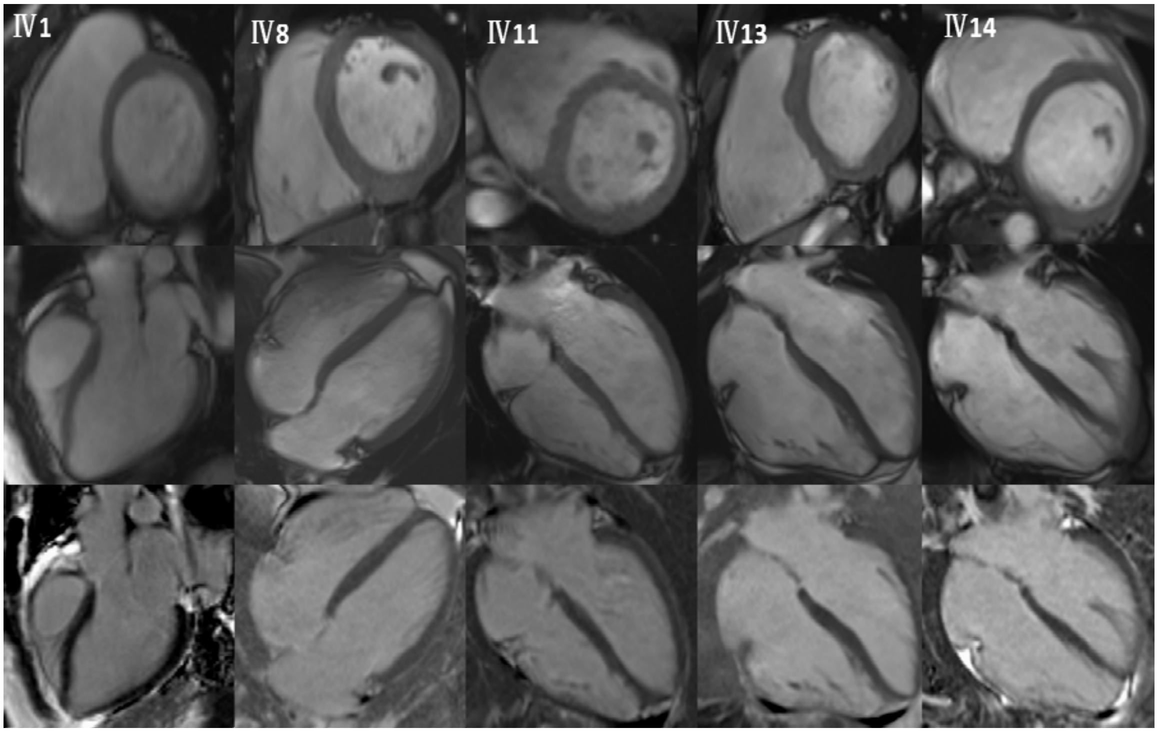

Supplement: Supplementary file 1 — Supplementary Information [file 41598_2018_19372_MOESM1_ESM.pdf]
